# General anesthesia with remimazolam in a patient with mitochondrial encephalomyopathy: a case report

**DOI:** 10.1186/s40981-021-00454-8

**Published:** 2021-06-23

**Authors:** Yuji Suzuki, Matsuyuki Doi, Yoshiki Nakajima

**Affiliations:** Department of Anesthesiology and Intensive Care Medicine, Hamamatsu University School of Medicine, 1-20-1 Handayama, Hamamatsu, Shizuoka, 431–3192 Japan

**Keywords:** Remimazolam, Mitochondrial encephalomyopathy with lactic acidosis and stroke-like episodes, Mitochondrial myopathy, Flumazenil

## Abstract

**Background:**

Systemic anesthetic management of patients with mitochondrial disease requires careful preoperative preparation to administer adequate anesthesia and address potential disease-related complications. The appropriate general anesthetic agents to use in these patients remain controversial.

**Case presentation:**

A 54-year-old woman (height, 145 cm; weight, 43 kg) diagnosed with mitochondrial encephalomyopathy with lactic acidosis and stroke-like episodes underwent elective cochlear implantation. Infusions of intravenous remimazolam and remifentanil guided by patient state index monitoring were used for anesthesia induction and maintenance. Neither lactic acidosis nor prolonged muscle relaxation occurred in the perioperative period. At the end of surgery, flumazenil was administered to antagonize sedation, which rapidly resulted in consciousness.

**Conclusions:**

Remimazolam administration and reversal with flumazenil were successfully used for general anesthesia in a patient with mitochondrial disease.

## Background

Mitochondrial diseases are caused by abnormalities in mitochondrial or nuclear genes that result in abnormal mitochondrial morphology or function [1, 2]. Cellular energy production is therefore disordered and organs with high energy demand are most affected [2]. Disease symptoms range from mild to severe and may involve multiple organs. Examples include ataxia, hearing loss, convulsions, cognitive decline, cardiomyopathy, myocardial conduction disorders, limb weakness, ophthalmoplegia, and diabetes [1, 3]. Systemic anesthetic management of patients with mitochondrial disease requires careful preoperative preparation to administer adequate anesthesia and address potential disease-related complications. However, clinical trials of anesthetic agents in adult mitochondrial myopathy patients have not been performed. The appropriate agents to use remain controversial [4].

Recently, remimazolam, an ultrashort-acting benzodiazepine, was approved for clinical use in Japan and is now available. Remimazolam is rapidly degraded by carboxylesterases in the liver and its metabolites have negligible pharmacological activity [5]. We report the successful use of intravenous remimazolam and its antagonist flumazenil for general anesthesia in a patient with mitochondrial myopathy.

## Case presentation

A 54-year-old woman (height, 145 cm; weight, 43 kg) diagnosed with mitochondrial encephalomyopathy with lactic acidosis and stroke-like episodes elected to undergo cochlear implantation to address her progressive bilateral sensorineural deafness. Preoperative electrocardiography showed no abnormalities. Transthoracic echocardiography showed circumferential thickening of the left ventricle and asymmetric hypertrophy of the septum, which was thicker than the posterior wall. Cardiac contractility was diffusely decreased and cardiac ejection fraction was 45%. Estimated glomerular filtration rate was low (27 mL/min) because of diabetic nephropathy. Although enhanced insulin therapy had been introduced, her HbA1c level was high (12.5%) immediately before surgery. Creatine kinase level was 162 IU/L (normal range, 41–153). Her medications included 5 mg oral imidapril daily and subcutaneous insulin (6 units in the morning, 8 units in the afternoon, and 6 units in the evening). She was not taking any antiepileptic drugs.

No premedication was administered. Intravenous remimazolam was administered as a 0.2 mg/kg bolus over 1 min, which resulted in loss of consciousness, followed by continuous infusion of remimazolam (1 mg/kg/h) and remifentanil (0.2 μg/kg/min). Neuromuscular monitoring of the left ulnar nerve was initiated using a train-of-four (TOF) stimulus (TOF watch SX®, MSD, Japan). Three minutes after administration of 30 mg of intravenous rocuronium, all four twitch responses disappeared and tracheal intubation was performed. During surgery, remimazolam was administered along with a continuous remifentanil infusion (0.2–0.25 μg/kg/min) to maintain the patient state index value between 25 and 50. Patient state index was measured using the SEDLine® monitor (Masimo Inc., Irvine, CA). A catheter was placed in the right radial artery for continuous arterial pressure monitoring. Intermittent blood gas analysis showed that the lactate concentration and pH ranged from 1.8 to 1.9 mmol/L and 7.41 to 7.45, respectively. Surgical time was 1 h and 34 min. Additional rocuronium administration was not needed throughout the surgery. After surgery was completed, the infusions were stopped and the TOF ratio was 0.91. The patient was extubated after stable spontaneous respirations with tidal volumes ≥ 8 mL/kg were confirmed (22 min after the end of surgery and 8 min after discontinuation of remimazolam). Thirteen minutes after extubation, her eyes remained closed; therefore, 200 μg of intravenous flumazenil was administered. Two minutes later, she opened her eyes, became verbally responsive, and was discharged from the operating room. After surgery, her only complaints were sore throat and nausea, which were treated with 1000 mg of acetaminophen and 10 mg of metoclopramide.

## Discussion

Selection of general anesthesia method is a concern in patients with mitochondrial disease [1]. Although the risk of malignant hyperthermia from inhalational anesthetics was previously believed to be increased in patients with muscular disease, this does not appear to be the case, even for patients with hereditary muscular disease. Malignant hyperthermia risk is not higher in patients with muscular dystrophy compared with the general population [6], and the same is assumed for patients with mitochondrial disease [7]. In fact, a survey of pediatric anesthesiologists in the USA showed that approximately 80% used sevoflurane to induce and maintain anesthesia in children with mitochondrial disease [7]. Propofol infusion syndrome is another concern because its risk is reportedly high in patients with mitochondrial disease [8]. Opinions vary regarding the selection of inhalational anesthetics or propofol. In our patient, we selected a benzodiazepine to avoid the risks of both. Benzodiazepines have been considered safe for general anesthesia in patients with mitochondrial disease and can be administered intravenously [7]. However, clearance is slow and prolonged effects may occur [9].

Remimazolam is an ultrashort-acting benzodiazepine with an imidazobenzodiazepine structure that exhibits pharmacological effects similar to those of midazolam. It has a side chain with an ester bond that is rapidly inactivated by carboxylesterase hydrolysis in the liver. The pharmacological activity of remimazolam metabolites is low, approximately 1/400th that of remimazolam, which suggests they have negligible pharmacological activity [5]. Furthermore, the effects of remimazolam can be reversed by flumazenil. These features make remimazolam a safe and feasible alternative to inhalational anesthetics and propofol.

The use of muscle relaxants in patients with mitochondrial disease presents potential difficulties. Patients with mitochondrial disease may experience muscle weakness and prolonged muscle relaxation after anesthesia. Muscle relaxant effects may be prolonged in these patients because of increased rocuronium sensitivity [10, 11]. To address this, sugammadex administration to reverse neuromuscular blockade may be useful, but experience is limited and no clear conclusion has yet been reached. In our patient, we did not administer sugammadex because the TOF ratio was ≥ 0.9 [12] and the effects of sugammadex in patients with muscular disease and TOF ratio ≥ 0.9 have not been investigated. Furthermore, sugammadex administration is associated with risks such as difficulty with reintubation and allergic reaction [13]. Therefore, the decision to use sugammadex should be considered carefully. Future studies are warranted.

Hearing impaired patients have difficulty perceiving changes in their environment and communicating, which are relevant to anesthesia recovery. Effective communication between the patient and medical personnel during recovery is crucial. Administration of flumazenil after remimazolam allows rapid psychomotor recovery from sedation [14], even in patients with muscular dystrophy [15]. The administration of flumazenil after remimazolam anesthesia may be useful to assist in achieving good communication with hearing impaired patients.

Remimazolam, remifentanil, and rocuronium can be pharmacologically antagonized, which is a major advantage in their use. The rapid and clear response achieved with flumazenil in our patient may indicate the superiority of remimazolam over other sedatives. However, flumazenil may cause convulsions by antagonizing benzodiazepine effects [16–18], and should not be administered casually.

## Conclusions

Administration of remimazolam, an ultrashort-acting benzodiazepine, and reversal with flumazenil enabled rapid and complete recovery from anesthesia in our patient with mitochondrial disease. Potential complications associated with flumazenil administration should be considered on an individual basis. Remimazolam is a feasible alternative to propofol and volatile anesthetics in patients with mitochondrial disease.

## Data Availability

Not applicable.

## Abbreviations

TOF: Train-of-four
